# Bilateral renal lymphangiectasia in a postpartum woman managed with renal decapsulation: a case report with follow-up after a subsequent pregnancy

**DOI:** 10.1186/s13256-026-06430-z

**Published:** 2026-08-06

**Authors:** André Torbey, Fares Kahal, Massa Johari, Sinan Abdulkader, Mazen Dughly

**Affiliations:** 1 Plastic Surgery Department, Glasgow Royal Infirmary, Glasgow, Scotland UK; 2 Faculty of Medicine, Syrian Private University, Damascus, Syria; 3 Damascus Hospital, Damascus, Syria; 4 New Medical Center Hospital, Damascus, Syria; 5 MRI Unit, Al Shifa Hospital, Damascus, Syria

**Keywords:** Renal lymphangiectasia, Postpartum period, Renal decapsulation, Bilateral renal disease, Perinephric fluid collection

## Abstract

**Background:**

Renal lymphangiectasia is a rare benign disorder involving the dilatation of renal lymphatic channels. While often incidental, it can cause significant morbidity through renal compression. We report a unique case of symptomatic bilateral disease presenting in the immediate postpartum period, managed with a novel surgical approach.

**Case presentation:**

A 23-year-old female presented five days postpartum with progressive left flank pain, nausea, and a blood pressure of 140/90 mmHg. Computed tomography (CT) demonstrated large bilateral perinephric fluid collections causing substantial compression of the kidneys and adjacent organs, along with moderate ascites. Fluid analysis from a perinephric drain was consistent with lymph.

**Interventions:**

Due to severe compressive effects and early recurrence following percutaneous drainage, the patient underwent laparoscopic surgical intervention. The procedure involved the complete excision of the markedly thickened renal capsule (decapsulation) and the creation of a posterior peritoneal drainage window to facilitate continuous fluid absorption.

**Conclusion:**

At 16 months post-operation, imaging confirmed significant radiological regression and preserved renal function. Notably, follow-up extended after a subsequent pregnancy showed no recurrence of fluid collections, and the patient remained asymptomatic. Complete renal decapsulation with a peritoneal window provided definitive treatment in this severe bilateral case.

## Background

Renal lymphangiectasia is a rare benign disorder caused by dilatation of renal lymphatic channels, leading to fluid accumulation around or within the kidney. Renal lymphangiectasia has also been described as renal lymphangiomatosis or renal lymphatic malformation. Its true prevalence is unknown, as the literature is largely limited to case reports and small case series. It has been reported in both children and adults, without a consistent age or sex predilection, and may be unilateral or bilateral with perinephric, peripelvic, intrarenal, or mixed involvement [1–4]. It may be incidental or present with flank pain, abdominal distension, hypertension, or renal dysfunction due to renal compression [5].

The pathogenesis is unclear and may be congenital, due to failed connection of renal lymphatics to central channels, or acquired from trauma, surgery, infection, or lymphatic obstruction [1,6].

Diagnosis relies on imaging, often using multiple modalities to distinguish it from conditions such as urinomas, abscesses, or cystic tumors. Aspiration typically yields sterile, lymphocyte-rich fluid with low creatinine [2,3,7,8].

Management is symptom-based, ranging from observation to drainage or surgical intervention in recurrent or severe cases [2,9].

Pregnancy-associated exacerbation of renal lymphangiectasia/lymphangiomatosis has been rarely reported; however, severe bilateral compressive disease presenting in the immediate postpartum period remains exceptionally uncommon. To our knowledge, management with complete renal decapsulation and creation of a peritoneal drainage window, followed by sustained clinical and radiological improvement after a subsequent pregnancy, has not been previously described [10].

We present the case of a 23-year-old woman who developed symptomatic bilateral perinephric fluid collections five days after delivery.

## Case presentation

A 23-year-old female presented with progressive left flank pain and nausea lasting two weeks, beginning five days postpartum. She denied fever or any other signs of infection. Her past medical history was notable only for urinary tract infections during her two previous pregnancies; she did not have a history of pre-eclampsia. The patient reported no significant family history of renal diseases or congenital urological anomalies. There was no other significant personal or family history. On examination, she appeared pale, and her blood pressure was 140/90 mmHg. Physical examination revealed tenderness localized to the left flank, extending toward the abdomen, without clinical signs of fluid thrill, shifting dullness, or peripheral/periorbital edema.

## Laboratory and imaging findings

Laboratory tests demonstrated anemia with hemoglobin at 8.0 g/dL and hematocrit of 26.7%. C-reactive protein (CRP) was elevated at 3.39 mg/dL. Renal function, electrolytes, serum albumin, and total protein were within normal limits. Urinalysis only demonstrated calcium oxalate crystals.

A left perinephric drain was placed under ultrasound guidance. Fluid analysis showed a creatinine level of 0.50 mg/dL, lymphocytic predominance, and biochemical features consistent with lymph. Cultures were negative.

CT scan identified a large left perinephric fluid collection measuring approximately 9 × 12.3 × 15.5 cm, causing substantial compression of the left kidney, spleen, pancreas, left adrenal gland, and adjacent bowel. Associated peripelvic fluid resulted in a characteristic ‘squashed’ appearance of the pelvicalyceal system, with severe narrowing of the renal pelvis and calyceal compression manifesting as early excretion. A smaller right perinephric fluid collection (approximately 3.2 × 8.4 cm) compressed the right kidney, right adrenal gland, and liver, accompanied by moderate peripelvic fluid causing severe stenosis of the renal pelvis and calyceal compression with early excretion.

**Fig. 1:**
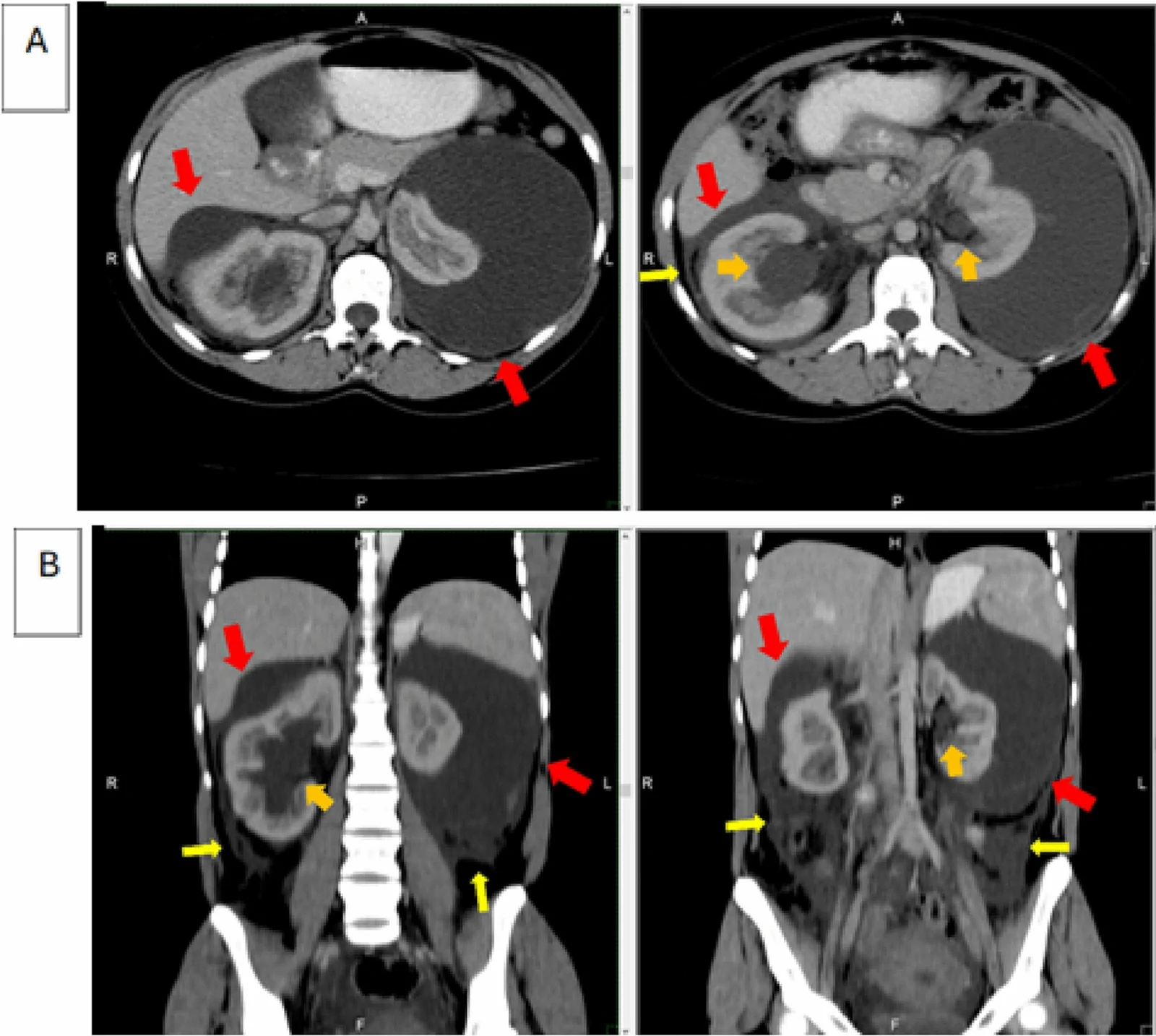
Axial (**A**) and coronal (**B**) contrast-enhanced computed tomography images of the abdomen during the portal venous phase demonstrating bilateral perinephric fluid collections (red arrows), peripelvic fluid collections (orange arrows), and ascites (yellow arrows). These findings are suggestive of bilateral peripelvic and perinephric renal lymphangiectasia, more pronounced on the left side, associated with moderate ascites

Moderate ascites was also noted throughout the abdomen and pelvis, along with a small umbilical hernia. Other abdominal organs appeared normal, and there was no radiographic evidence of renal vein thrombosis or other vascular pathology. No lymphadenopathy was detected. Tiny vascular calcifications were seen in the left pelvic vessels. Lung bases were clear (Fig. 1).

Lymphoscintigraphy was not utilized, as the clinical presentation, pathognomonic CT findings, and fluid analysis were diagnostic.

## Management

Surgery was suggested due to severe compressive effects, persistent symptoms, and inadequate response to drainage alone. While the disease was bilateral, the right-sided collection was managed conservatively. Surgical intervention was prioritized for the left kidney as it was the primary source of the patient’s severe, acute symptoms and exhibited a much larger perinephric fluid collection.

Under general anesthesia, pneumoperitoneum was established via a supraumbilical port, and three additional trocars were inserted: epigastric, left lower quadrant (opposite McBurney’s point), and infraumbilical midline. The patient was positioned in a slight left-up tilt.

The left colon was mobilized by dissecting along the line of Toldt to expose the retroperitoneum, where a large accumulation of lymphatic fluid was identified beneath a significantly thickened renal capsule. Due to the degree of fibrotic thickening, the entire renal capsule was excised *en bloc* rather than simply drained, and the specimen was sent for histopathological analysis which revealed a fibrous capsule containing blood vessels, hemorrhage, and mild chronic inflammation. No epithelial lining or renal tissue was identified, and no evidence of neoplasia was seen.

Additionally, a portion of the posterior peritoneal wall was excised to create a drainage window between the retroperitoneal and peritoneal cavities to facilitate lymphatic fluid absorption. A Jackson–Pratt drain was placed through the window. Hemostasis was confirmed, and the fluid was aspirated.

The right-sided collection was monitored with a wait-and-see approach, with plans for intervention if clinical or radiological status did not improve.

## Follow-up and outcome

At one-month post-procedure, her blood pressure was 110/80 mmHg, and ultrasound showed reduced bilateral perinephric fluid, with persistent right hydronephrosis. At six months, she remained asymptomatic, with further reduction in fluid collections and improved right hydronephrosis.

At 16 months post-operation, CT showed near-complete resolution of the left perinephric collection, with a small residual left peripelvic collection (16 × 27 × 36 mm, 10 HU) causing severe pelvic stenosis and calyceal compression.

On the right, a small perinephric collection (16 mm, 11 HU) and moderate peripelvic fluid caused severe pelvic narrowing and calyceal compression with early excretion.

Minimal ascites and a small umbilical hernia were noted; all other organs were normal.

Throughout follow-up, serum creatinine and estimated glomerular filtration rate (eGFR) remained normal, confirming preserved renal function, despite the persistence of the residual peripelvic collections.

**Fig. 2:**
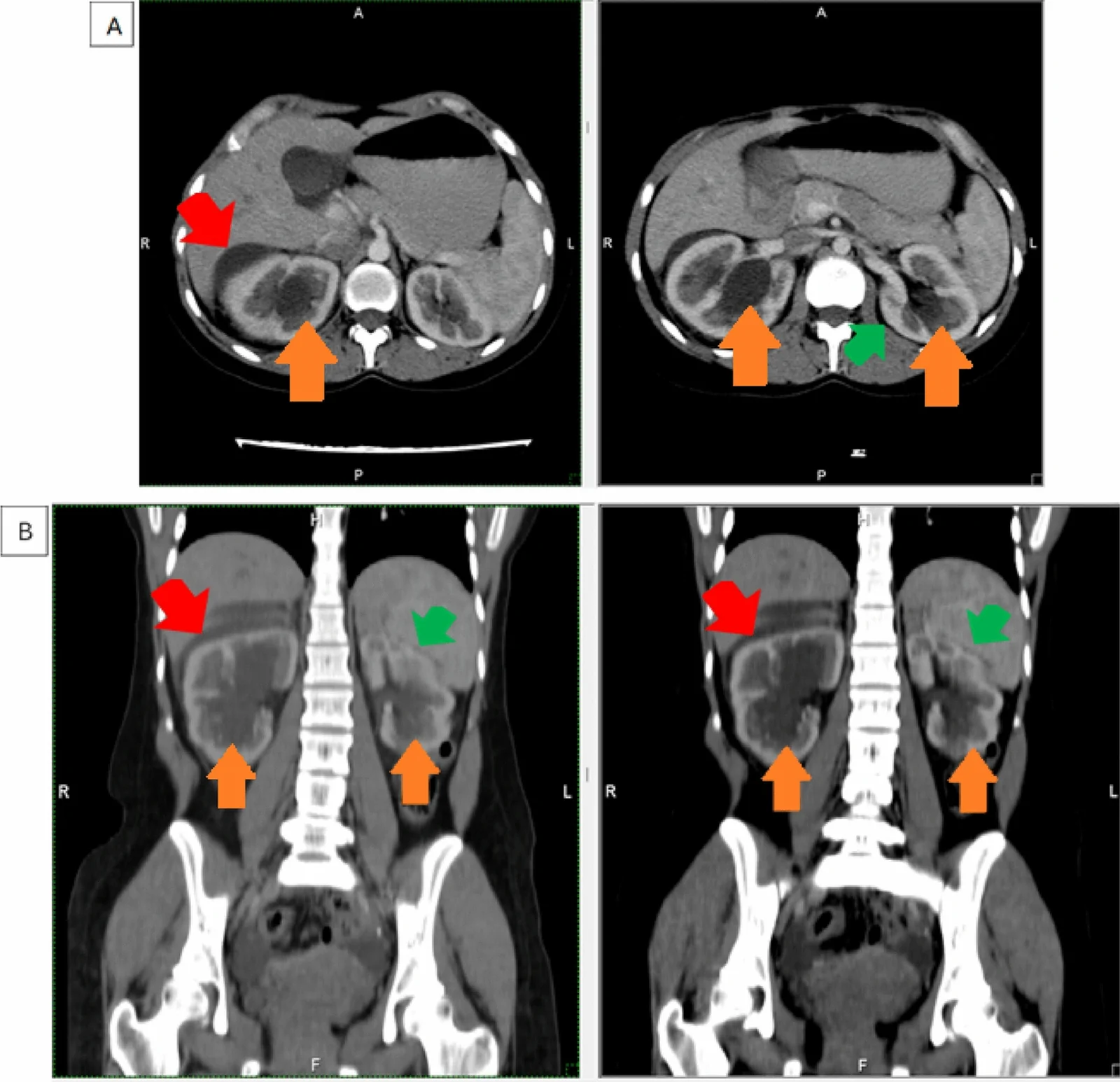
Axial (**A**) and coronal (**B**) contrast-enhanced computed tomography images of the abdomen in the portal venous phase, obtained approximately 16 months post-operation, demonstrate minimal residual right perinephric fluid collections (red arrows) and near-complete resolution of the left perinephric collection (green arrows). Persistent peripelvic fluid collections (orange arrows). These findings are consistent with bilateral peripelvic and perinephric renal lymphangiectasia, showing significant improvement compared to the previous study

Overall, findings indicate improving bilateral peripelvic and perinephric renal lymphangiectasia, with marked resolution of perinephric collections and stable peripelvic involvement (Fig. 2).

## Second pregnancy and consequent follow-up

At 19 months post-operation, the patient reported a subsequent pregnancy. Given that her initial presentation was temporally associated with the postpartum period, follow-up was intentionally extended. A postpartum ultrasound performed two months after delivery demonstrated no recurrence of perinephric or peripelvic fluid collections, and the patient remained asymptomatic.

## Discussion

Renal lymphangiectasia is typically unilateral, asymptomatic, and often discovered incidentally. Although usually benign, the clinical spectrum is broad. Reported presentations include flank or abdominal pain, abdominal distension, ascites, haematuria, hypertension, proteinuria, renal dysfunction, and rarely renal vein thrombosis. Radiologically, the condition may mimic hydronephrosis, parapelvic cysts, polycystic kidney disease, urinoma, abscess, or cystic renal neoplasm, particularly when peripelvic collections compress the collecting system [1–5].

In contrast, our case involved symptomatic bilateral disease with recurrent perinephric fluid accumulation requiring surgical intervention. Its onset in the immediate postpartum period is also rarely described in the literature, suggesting a possible role for transient physiological changes, such as increased intra-abdominal pressure, blood volume, and hormonal levels, in triggering lymphatic congestion and revealing underlying congenital anomalies [1,6].

Pregnancy-related cases remain rare. Özmen et al. described exacerbation of renal lymphangiomatosis during pregnancy, managed with percutaneous drainage until delivery. Other bilateral cases have been reported outside pregnancy, with variable clinical courses ranging from incidental or conservatively managed disease to symptomatic cases requiring drainage, sclerotherapy, marsupialization, or surgical decompression. This variability reflects the absence of a standardized treatment pathway and supports tailoring management to symptom burden, renal compression, recurrence, and renal function [1,3,4,10].

Management of this condition remains non-standardized. Asymptomatic cases may be monitored, while symptomatic or recurrent presentations often require intervention. Percutaneous drainage, though rarely curative, can aid in diagnosis and symptom relief, but typically results in recurrence unless continuous decompression is maintained [9].

Surgical options include marsupialization, deroofing, and, in severe or recurrent cases, nephrectomy. The choice depends on the extent of lymphatic involvement and complications such as renal vein thrombosis or renal impairment. For instance, marsupialization, which is creating a permanent opening for ongoing lymphatic drainage, is used when dilations are localized, while nephrectomy may be necessary in cases with significant vein involvement or renal failure [1,7,9].

In our patient, the extent of bilateral disease, significant renal compression, and early recurrence following percutaneous drainage prompted definitive surgical management to prevent long-term hypertensive sequelae and preserve nephron function. Unlike simple drainage or deroofing described in many reports, we performed complete excision of the markedly thickened renal capsule combined with creation of a peritoneal drainage window. Decapsulation was selected because drainage had already resulted in early recurrence, and intraoperatively the collection was associated with a markedly thickened fibrotic renal capsule rather than a discrete cyst suitable for focal deroofing or marsupialization. Complete decapsulation allowed wider decompression while preserving renal parenchyma and avoiding nephrectomy. The peritoneal window was created to provide ongoing low-pressure drainage. Potential risks include bleeding, renal or collecting-system injury, infection, persistent lymphatic leakage, recurrence, and need for further intervention. This definitive approach was necessitated by the diffuse, multiloculated nature of the disease, as complete capsular excision rendered focal marsupialization anatomically unfeasible. This resulted in sustained clinical improvement, marked radiological regression of perinephric collections, and preservation of renal function on long-term follow-up, even after a second pregnancy, despite the initial presentation being temporally associated with the postpartum period, supporting its role in selected severe cases refractory to conservative measures. We suspect the preoperative hypertension was driven by a Page kidney mechanism secondary to extrinsic renal compression, as evidenced by the marked radiological compression and normalization of blood pressure following surgical decompression. Although preoperative plasma renin activity was not measured, these findings strongly support the diagnosis.

## Conclusion

This case highlights several important considerations. Renal lymphangiectasia should be considered in postpartum patients with flank pain and perinephric fluid collections, especially when imaging is characteristic. Percutaneous drainage is useful for diagnosis, but early surgical intervention should be considered in cases with bilateral involvement, recurrent collections, or compressive symptoms. Complete renal decapsulation with a peritoneal drainage window provided definitive treatment in this patient, leading to sustained clinical improvement, significant radiological regression, and preserved renal function even after a second pregnancy.

## Data Availability

No datasets were generated or analyzed during the current study.

## Abbreviations

CRP: C-reactive protein
CT: Computed tomography
HU: Hounsfield units
